# Webcams, Crowdsourcing, and Enhanced Crosswalks: Developing a Novel Method to Analyze Active Transportation

**DOI:** 10.3389/fpubh.2016.00097

**Published:** 2016-05-19

**Authors:** J. Aaron Hipp, Alicia Manteiga, Amanda Burgess, Abby Stylianou, Robert Pless

**Affiliations:** ^1^Department of Parks, Recreation, and Tourism Management, Center for Geospatial Analytics, North Carolina State University, Raleigh, NC, USA; ^2^Prevention Research Center, Washington University in St. Louis, St. Louis, MO, USA; ^3^Department of Computer Science and Engineering, Washington University in St. Louis, St. Louis, MO, USA

**Keywords:** crowdsourcing, active transportation, webcams, built environment, crosswalks, pedestrian detection, bicyclist detection

## Abstract

**Introduction:**

Active transportation opportunities and infrastructure are an important component of a community’s design, livability, and health. Features of the built environment influence active transportation, but objective study of the natural experiment effects of built environment improvements on active transportation is challenging. The purpose of this study was to develop and present a novel method of active transportation research using webcams and crowdsourcing, and to determine if crosswalk enhancement was associated with changes in active transportation rates, including across a variety of weather conditions.

**Methods:**

The 20,529 publicly available webcam images from two street intersections in Washington, DC, USA were used to examine the impact of an improved crosswalk on active transportation. A crowdsource, Amazon Mechanical Turk, annotated image data. Temperature data were collected from the National Oceanic and Atmospheric Administration, and precipitation data were annotated from images by trained research assistants.

**Results:**

Summary analyses demonstrated slight, bi-directional differences in the percent of images with pedestrians and bicyclists captured before and after the enhancement of the crosswalks. Chi-square analyses revealed these changes were not significant. In general, pedestrian presence increased in images captured during moderate temperatures compared to images captured during hot or cold temperatures. Chi-square analyses indicated the crosswalk improvement may have encouraged walking and biking in uncomfortable outdoor conditions (*P* < 0.5).

**Conclusion:**

The methods employed provide an objective, cost-effective alternative to traditional means of examining the effects of built environment changes on active transportation. The use of webcams to collect active transportation data has applications for community policymakers, planners, and health professionals. Future research will work to validate this method in a variety of settings as well as across different built environment and community policy initiatives.

## Introduction

Active transportation, bicycling and walking between destinations, is associated with reduced rates of chronic disease and the promotion of healthier lifestyles in comparison to vehicle trips (1–5). Some characteristics of the built environment influence the adoption of behavioral changes, encouraging individuals to choose walking or biking over personal motor vehicle use (1–3, 5–7). Characteristics, such as perceived safety, proximity to destinations, the presence of foliage and green spaces, and traffic control features, such as stop signs and speed bumps, can be positive-mediating factors in this decision-making process (1–3, 5–9). However, there is little agreement on specific infrastructural improvements that lead to increases in biking or walking (9).

Pedestrian safety has been at the forefront of a body of research evaluating built environment characteristics that aid or hinder the decision to walk (10–12). Most crosswalk enhancement studies focus on reducing pedestrian and vehicular collisions (10–12). Basic marked crosswalks are more effective than unmarked crosswalks at increasing pedestrian safety. Evidence indicates stand-alone crosswalks, independent of other interventions, such as speed limit reductions or speed bumps, reduced the number of intersection collisions across 30 cities in the United States (10). Beyond pedestrian safety, few studies have analyzed the effects of adding or enhancing crosswalks on pedestrian activity.

Permanent built environment features, such as bicycle boulevards and improved bike lanes, are associated with an increase in biking (7, 13–15). The relationship between factors influencing the use of biking spaces is complex, making it difficult to measure the effects of this infrastructure change on active transportation (7, 16).

The decision to choose to walk or bike instead of drive is associated with weather as well as with features of the built environment (4, 6, 17, 18). Pedestrian activity decreases overall during snow, ice, and cold temperatures in winter seasons, but individuals are more likely to choose walking over biking when the temperature is cooler (4, 17–19). There is a lack of literature on the role temperature plays in the utilization of new built environment features in a community, which is likely due to the complex nature of the data needed to study such outcomes.

Understanding trends in pedestrian and bicyclist behaviors, especially in response to specific built environment interventions, allows key stakeholders to select policies for implementation that will have the greatest impact on their community’s specific active transportation needs (4, 9, 20).

The evaluation of built environment interventions requires active monitoring of these changes in outdoor spaces (4, 5, 9, 13, 20). The Archive of Many Outdoor Scenes (AMOS^1^) is a database that has compiled images captured by publicly available webcams since 2006 (e.g., traffic cameras) (14, 21). Images gathered from the AMOS database (over 885 million as of March 2016) can be analyzed to study changes in the built environment as well as associated changes in active transportation. The use of webcams to study active transportation can provide researchers and practitioners access to outcome data regarding the impact of built environment enhancements on active transportation (15, 22).

The manual annotation of a large number of webcam images has the potential to be both time consuming and costly, but emerging technologies, such as crowdsourcing, can alleviate these constraints (23–25). Amazon’s Mechanical Turk (MTurk^2^) is a crowdsourcing platform that allows anyone to design a human intelligence task (HIT), such as counting people in webcam images, and then post this HIT online for individuals over the age of 18 to complete *via* the internet (23, 25). Posting a HIT to the “crowd” of human workers online allows researchers to obtain quality data quickly and inexpensively (23–25).

This case study builds on previous research, which demonstrated the method of using webcams and crowdsourcing can be used to reliably, validly, and inexpensively monitor trends in active transportation (22). The objectives of this case study were as follows: (1) to determine if trends in active transportation are influenced by a change in the built environment, (2) to explore interactions between built environment enhancements, weather conditions, and active transportation, and (3) to further develop a novel method for objectively and inexpensively measuring the effects of built environment changes on active transportation.

## Materials and Methods

### Study Sample

**Figure 1:**
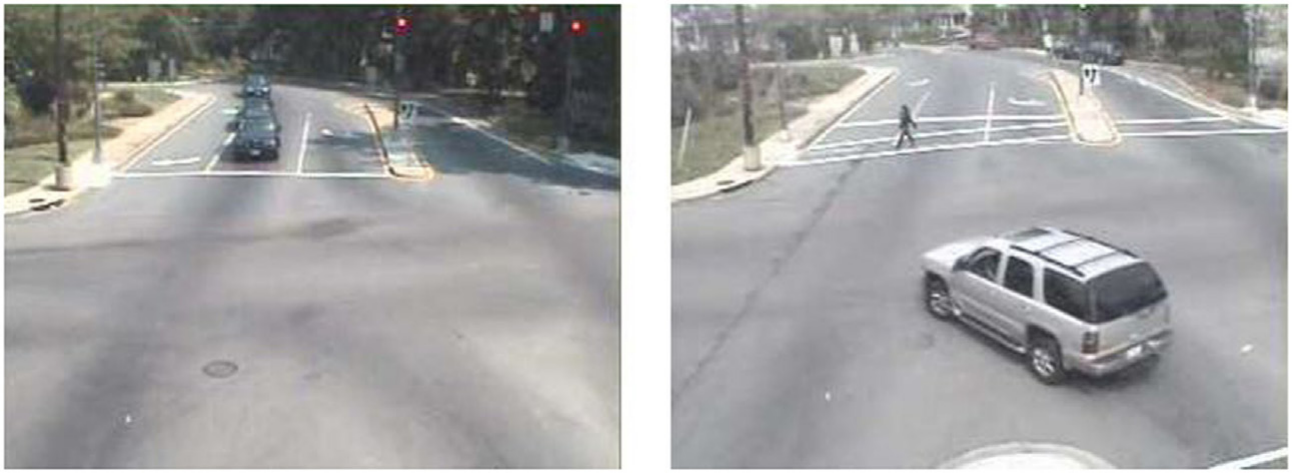
**Residential intersection prior to (left) and following (right) built environment change**.

**Figure 2:**
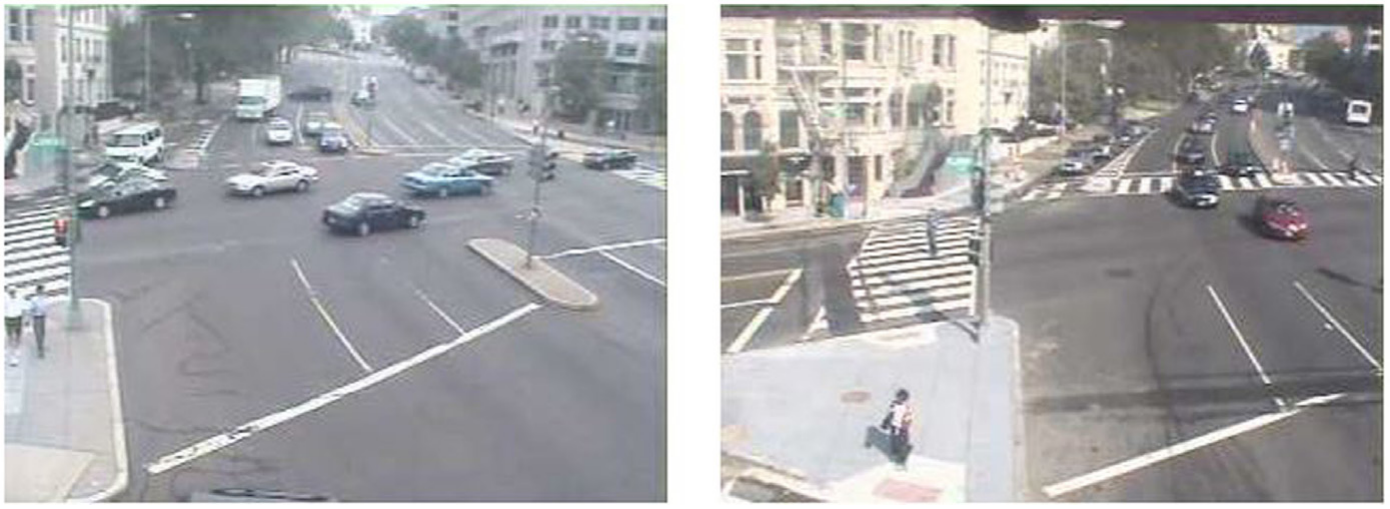
**Commercial intersection prior to (left) and following (right) built environment change**.

Active transportation data were from two webcams captured by the AMOS dataset. Webcams used in this study are located at the intersections of Piney Branch Road NW and Eastern Avenue NW in Washington, DC, USA, 20012 (residential area), and Connecticut Avenue NW and Florida Avenue NW in Washington, DC, USA, 20009 (commercial area). The two intersection webcams were selected because they have a clear view of pedestrians, bicyclists, and vehicles, and because both captured the enhancement of crosswalks on November 20, 2007 (Figures 1 and 2). At the residential intersections, a standard crosswalk was painted across a four-lane road where previously there was no crosswalk. At the commercial intersection, a faded crosswalk on a seven-lane road was re-painted and upgraded from a standard crosswalk to a more visible ladder crosswalk. Intersections were classified for general land use using Google Street View (26, 27).

The AMOS dataset captures and archives an image every 30 min (48 images per day) once the specific webcam is added to the dataset by a researcher or the public.^3^ For the residential webcam,^4^ images were collected and archived between May 7, 2007 and July 2, 2015. For the commercial webcam,^5^ images were collected and archived between May 7, 2007 and January 7, 2015. Using these two AMOS datasets, a total of 20,529 webcam images were downloaded for annotation representing daylight images between May 7 and November 19, 2007, and between May 7 and November 19, 2008.

To examine the effects of temperature and precipitation on the use of crosswalks, images were matched with an hourly average temperature and precipitation status. Due to limited data availability, researchers could only combine 8,067 (39%) images with hourly temperature data and elected to use crowdsource workers to annotate if precipitation was present in the captured image (e.g., wet roads). Temperature data were collected from the National Oceanic and Atmospheric Administration: National Centers for Environmental Information.^6^ These hourly, citywide temperatures were collected at the Washington National Airport, located 12 and 7 miles away from the residential and commercial intersections, respectively.

The Amazon Mechanical Turk (MTurk) website was used as the crowdsourcing platform to annotate the number of pedestrians and bicyclists in each captured image, as well as presence of precipitation. Each image was annotated by four unique MTurk workers, the minimum number found to be both reliable and valid (15). MTurk workers were paid US $0.02 per image between September and December 2013. Prompts MTurk workers responded to included

1. Please outline each bicycle or person riding a bicycle in the scene.
2. Please outline each pedestrian in the scene.
3. What is the weather in this image? Sunny, Cloudy, Rainy, or Snowy.

Each image was annotated at least four unique times (*N* = 82,116), for a total cost of $1,642.32.

Counts per transportation mode were downloaded to SPSSv.22 (IBM, Chicago) for analysis in March 2015.

### Statistical Analyses

Summaries of weekday, weekend, and overall presence of pedestrians and bicyclists, regardless of crosswalk improvements, were calculated at both intersections, and have been reported elsewhere (22). Chi-square analyses were performed to study differences in pedestrian and bicyclist presence in images before and after crosswalk enhancement.

Average and SDs of collected temperature data were calculated. Scatterplots were created to visually portray the relative frequency of pedestrians per intersection across temperatures. Temperatures were divided into “normal” (within 1 SD of the May–November mean temperature) and “non-normal” (outside 1 SD of the mean temperature) categories. In calculating “Climate Normals,” NOAA presents averages and SDs (28). Summaries of the number of images with pedestrians and bicyclists, prior to and following crosswalk enhancement, at normal and non-normal temperatures were calculated. Chi-square analyses were performed to determine if there were differences in bicyclist and pedestrian presence when temperatures were normal versus non-normal.

Descriptive summaries of the number of images MTurk workers identified as rainy were compiled to determine the degree of agreement between crowdsource workers. Research assistants identified all images as showing signs of precipitation (wet ground and rain) or not. Descriptive summaries of the number of images with pedestrians and bicyclists, prior to and following crosswalk enhancement, in images with signs of precipitation and without them, were calculated. Chi-square analyses were performed to determine if there were differences in bicyclist and pedestrian presence when there was precipitation detected by research assistants versus when there was not.

## Results

### Descriptive Statistics

At the residential intersection, 4,959 images were captured prior to and 5,007 images were captured following the crosswalk enhancement. Pedestrians were present in 298 images (6.0%) captured prior to the change and in 337 images (6.7%) captured following the change. Bicyclists were present in 79 images (1.6%) captured before the change, and in 86 images (1.7%) captured after the change.

At the commercial intersection, 5,246 images were captured prior to the crosswalk enhancement and 5,317 images were captured following the crosswalk enhancement. Pedestrians were annotated in 3,658 images (69.7%) captured prior to the change, and in 3,615 images (68.0%) captured following the change. Bicyclists were annotated in 581 images (11.1%) captured before the change, and in 565 images (10.6%) captured after the change (Table 1).

**Table 1:** **Number of images with pedestrians and bicyclists before and after crosswalk enhancement**.

Outcome	Number of images	Images with pedestrians or bicyclists^a^ (%)	Pre-crosswalk: number of images with pedestrians or bicyclists (%)	Post-crosswalk: number of images with pedestrians or bicyclists (%)	*P* value (*X*^2^)^b^
**Residential**					
Pedestrians	9,966	635 (6.37)	298 (6.01)	337 (6.73)	0.14
Weekday	7,231	500 (6.91)	233 (6.19)	267 (7.36)	0.14
Weekend	2,735	135 (4.94)	65 (4.79)	70 (5.08)	0.73
Bicyclists	9,966	135 (1.35)	79 (1.59)	86 (1.72)	0.63
Weekday	7,231	120 (1.66)	50 (1.39)	70 (1.93)	0.07
Weekend	2,735	45 (1.65)	29 (2.14)	16 (1.16)	**0.04**
**Commercial**					
Pedestrians	10,563	7,273 (68.85)	3,658 (69.73)	3,615 (67.99)	0.05
Weekday	7,599	5,412 (71.22)	2,727 (72.28)	2,685 (70.18)	**0.04**
Weekend	2,964	1,861 (62.79)	931 (63.20)	930 (62.37)	0.64
Bicyclists	10,563	1,146 (10.85)	581 (11.08)	565 (10.63)	0.46
Weekday	7,599	844 (11.11)	439 (11.64)	405 (10.59)	0.15
Weekend	2,964	302 (10.19)	142 (9.64)	160 (10.73)	0.33

*^a^Refers to the sum of relevant images captured prior to and following the crosswalk enhancement*.

*^b^Refers to the significance of association between presence of bicyclists/pedestrians before and after the crosswalk enhancement. A *P* value <0.05 is considered significant*.

*Boldface indicates statistical significance, *P* < 0.05*.

**Figure 3:**
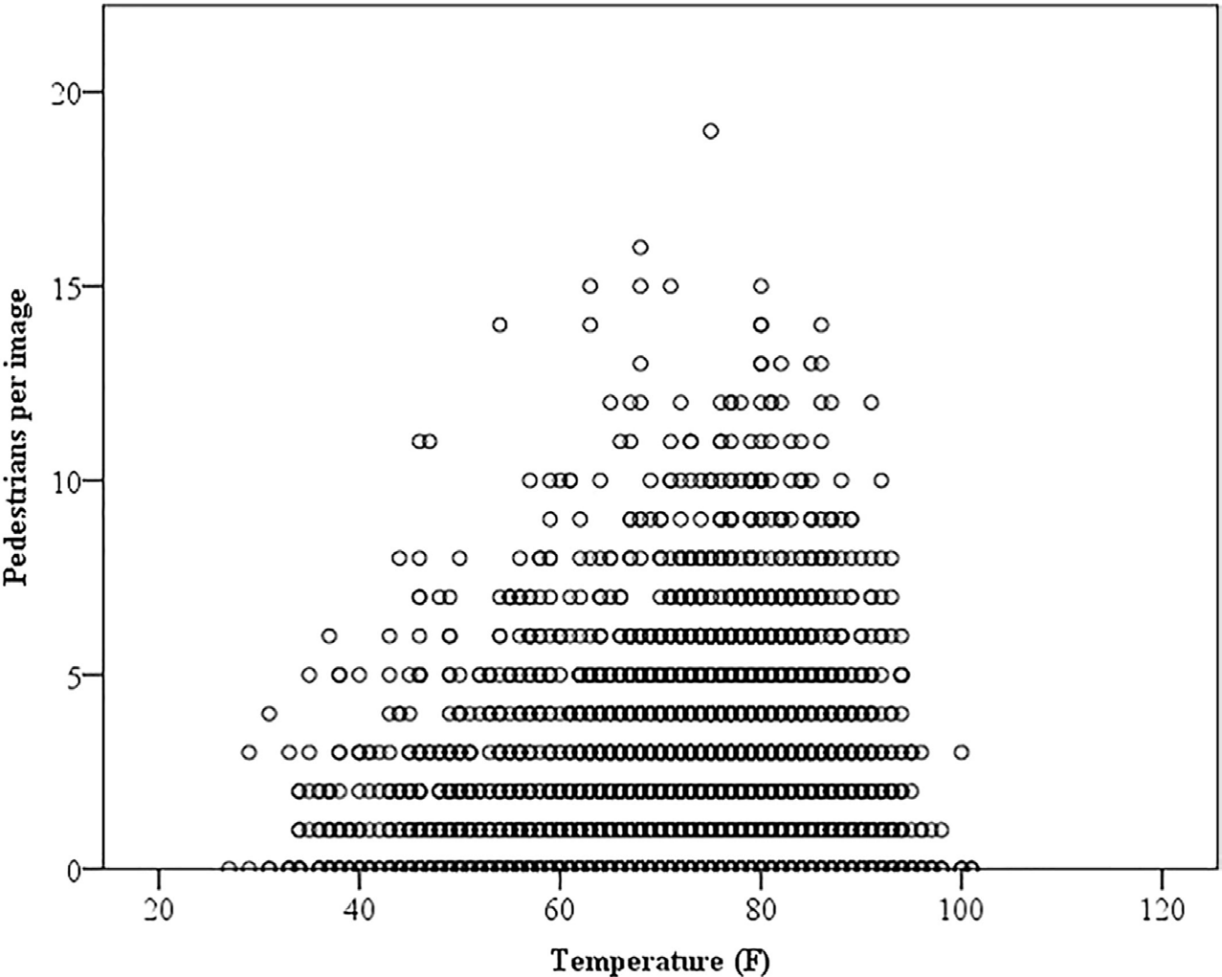
**Number of pedestrians per image across temperature**.

The 3,883 images at the residential intersection and 4,184 images at the commercial intersection were matched with temperature data. Temperatures ranged from 27 to 101°F, with an average of 74°, and a SD of 12°. In general, there were more pedestrians per image when temperatures were within 1 SD of the mean (between 62° and 86°) (Figure 3). Bicyclist annotation patterns were not related to temperature at both intersections (Table 2).

**Table 2:** **Pedestrian and bicyclist presence at various temperatures, by intersection**.

Temperature^a^	Transportation mode	Number of images	Number of images with pedestrians/bicyclists (%)
**Residential**			
<50	Pedestrians	238	24 (10.08)
	Bicyclists		1 (0.42)
50–62	Pedestrians	395	26 (6.58)
	Bicyclists		7 (1.77)
62–86^b^	Pedestrians	2,747	226 (8.23)
	Bicyclists		54 (1.97)
86–98	Pedestrians	499	37 (7.41)
	Bicyclists		17 (3.41)
>98	Pedestrians	4	0 (0.00)
	Bicyclists		0 (0.00)
**Commercial**			
<50	Pedestrians	255	168 (65.88)
	Bicyclists		26 (10.20)
50–62	Pedestrians	419	289 (68.97)
	Bicyclists		57 (13.60)
62–86^b^	Pedestrians	2,961	2,135 (72.10)
	Bicyclists		376 (12.70)
86–98	Pedestrians	545	392 (71.93)
	Bicyclists		61 (11.19)
>98	Pedestrians	4	1 (25.00)
	Bicyclists		1 (25.00)

*^a^Temperatures reported in degrees Fahrenheit*.

*^b^Normal temperature range – within 1 SD of average temp*.

Temperatures were divided into two categories: non-normal (<62° or >86°) and normal (between 62° and 86°, inclusive). At the residential intersection, before the enhancement of the crosswalk and when temperatures were non-normal, 32 images (6.6%) included pedestrians. After the enhancement when temperatures were non-normal, 55 images (8.4%) included pedestrians. There were 10 images (2.1%) and 13 images (2.0%) with Bicyclists before and after the change, respectively, when temperatures were non-normal.

At the commercial intersection prior to the crosswalk enhancement and when temperatures were non-normal, 373 images (67.7%) included pedestrians. This changed to 477 images (66.5%) after the change. Bicyclist annotation in images captured during periods of non-normal temperatures changed from 67 images (12.6%) to 78 images (11.6%) after the crosswalk enhancement (Table 3).

**Table 3:** **Number of images with pedestrians and bicyclists before and after crosswalk enhancement, in normal versus non-normal temperatures**.

Transportation mode	Pre-/post-crosswalk change	Number of images	Images with pedestrians/bicyclists (%), normal temperature^a^	Images with pedestrians/bicyclists (%), non-normal temperature^b^	*P* value (*X*^2^)^c^
**Residential**					
Pedestrians	Pre	1,895	111 (7.87)	32 (6.61)	0.37
	Post	1,988	115 (8.61)	55 (8.44)	0.90
	*P* value (*X*^2^)^d^		0.21	0.25	
Bicyclists	Pre	1,895	23 (1.63)	10 (2.07)	0.23
	Post	1,988	31 (2.32)	13 (1.99)	0.64
	*P* value (*X*^2^)^d^		0.76	0.51	
**Commercial**					
Pedestrians	Pre	2,064	1,093 (72.24)	373 (67.70)	**0.04**
	Post	2,120	1,042 (71.96)	477 (66.52)	0.64
	*P* value (*X*^2^)^d^		0.86	0.21	
Bicyclists	Pre	2,064	193 (12.76)	67 (12.16)	0.72
	Post	2,120	183 (12.64)	78 (11.61)	0.50
	*P* value (*X*^2^)^d^		0.92	0.76	

*^a^“Normal” temperatures between 62 and 86 are within 1 SD of the average temperature*.

*^b^“Non-normal” temperatures below 62 and above 86 are outside 1 SD of the average temperature*.

*^c^Refers to the significance of association between presence of bicyclists/pedestrians at normal versus non-normal temperatures. A *P* value <0.05 is considered significant*.

*^d^Refers to the significance of association between presence of bicyclists/pedestrians before versus after the crosswalk enhancement. A *P* value <0.05 is considered significant*.

*Boldface indicates statistical significance *P* < 0.05*.

Researchers attempted to assess precipitation by asking MTurk workers the following question: what is the weather in this image: Sunny, Cloudy, Rainy, Snowy. These four options were collapsed into two categories, no precipitation or precipitation. Across only 55% of all images did all four MTurk workers agree on the image having precipitation or not. Therefore, researchers determined the wording of the question was not reliable.

Trained research assistants then assessed 19,665 images for precipitation. At the residential intersection, prior to crosswalk enhancement and in images in which research assistants detected precipitation (340 images total), 16 images (4.7%) included pedestrians and only 1 (0.3%) included bicyclists (Table 4). Under these same conditions (residential intersection, precipitation) following crosswalk enhancement, 17 (4.5%) of the sample of 378 images included pedestrians and 5 (1.3%) included bicyclists.

**Table 4:** **Images with pedestrians and bicyclists before and after crosswalk enhancement, with and without precipitation**.

Transportation mode	Pre-/post-crosswalk change	*N* images	Images with pedestrians/bicyclists	Images with pedestrians/bicyclists (%), no precipitation	Images with pedestrians/bicyclists (%), precipitation	*P* value (*X*^2^)^a^
**Residential**						
Pedestrians	Pre	4,538	288	272 (6.5%)	16 (4.7% of all pre-BE change images captured during precipitation)	**0.01**
	Post	4,654	297	280 (6.5%)	17 (4.5%)	**0.01**
	Total	9,192	585	552 (6.5%)	33 (4.6%)	
	*P* value (*X*^2^)^b^			0.898	0.894	
Bicyclists	Pre	4,538	76	75 (1.8%)	1 (0.3%)	**0.04**
	Post	4,654	79	74 (1.7%)	5 (1.3%)	0.78
	Total	9,192	155	149 (1.8%)	6 (0.8%)	
	*P* value (*X*^2^)^b^			0.845	0.131	
**Commercial**						
Pedestrians	Pre	5,196	3,658	3,501 (70.7%)	157 (63.8%)	**0.00**
	Post	5,277	3,605	3,411 (69.2%)	194 (56.1%)	**0.00**
	Total	10,473	7,263 (*X*%)	6,912 (70.7%)	351 (59.3%)	
	*P* value (*X*^2^)^b^			0.092	0.059	
Bicyclists	Pre	5,196	581	564 (11.4%)	17 (6.9%)	**0.00**
	Post	5,277	564	540 (11.0%)	24 (6.9%)	**0.02**
	Total	10,473	1,145 (*X*%)	1,104 (11.2%)	41 (6.9%)	
	*P* value (*X*^2^)^b^			0.485	0.990	

*^a^Refers to the significance of association between presence of bicyclists/pedestrians when precipitation was observed in images versus when no precipitation was observed. A *P* value of <0.05 is considered significant*.

*^b^Refers to the significance of association between presence of bicyclists/pedestrians before versus after the crosswalk enhancement*.

*Boldface indicates statistical significance, *P* < 0.05*.

At the commercial intersection, prior to crosswalk enhancement in images with precipitation (246 total), 157 (63.8%) of images included pedestrians and 17 images (6.9%) included bicyclists. Following crosswalk enhancement (sample size of 346 images), 194 (56.1%) of images included pedestrians and 24 (6.9%) included bicyclists when precipitation was detected.

### Chi-Square Analyses

Chi-square tests of independence were performed to examine the relationship between presence of pedestrians and bicyclists in images before and after the enhancement of crosswalks (Table 1). The overall (weekday and weekends combined) relationship between pedestrian presence and crosswalk enhancement at the residential intersection was not significant. The overall relationship between bicyclist annotation and crosswalk enhancement was also not significant, though there was a significant decrease in bicyclist annotation during weekends after the crosswalk enhancement [*X*^2^(1, *N* = 2,735) = 4.04, *P* = 0.04]. At the commercial intersection, the relationships between crosswalk enhancement and pedestrian presence, and crosswalk enhancement and bicyclist presence were not significant overall. However, there was a significant decrease in pedestrian presence during weekdays after the crosswalk enhancement [*X*^2^(1, *N* = 7,599) = 4.08, *P* = 0.04].

Chi-square tests of independence were then performed to examine the relationship between presence of pedestrians and bicyclists in images before and after the enhancement of crosswalks at both normal and non-normal temperatures (Table 3). At the residential intersection prior to the crosswalk enhancement, there was no relationship between pedestrian annotation and temperature or between pedestrian annotation and temperature after the improvement of the crosswalk. There was no relationship between bicyclist annotation and temperature, both prior to and following the crosswalk enhancement.

At the commercial intersection prior to the crosswalk enhancement, there were significantly more images with a pedestrian present during normal temperatures than during non-normal temperatures [*X*^2^(1, *N* = 2,064) = 4.06, *P* < 0.05]. However, after the completion of the crosswalk enhancement, there was no significant relationship between pedestrian presence and temperature [*X*^2^(1, *N* = 2,120) = 0.22, *P* > 0.05]. There was no relationship between bicyclist annotation and temperature, both prior to and following the crosswalk enhancement.

Finally, chi-square tests of independence were performed to examine the relationship between presence of pedestrians and bicyclists in images before and after the enhancement of crosswalks in images with and without visible signs of precipitation (Table 4). Not surprisingly, there was a relationship between both pedestrian and bicyclist presence and precipitation. This relationship existed at both intersections, both prior to and following crosswalk enhancement. However, there was no significant relationship between bike presence and precipitation following the crosswalk enhancement at the residential intersection [before: *X*^2^(1, *N* = *Z*) = *X*, *P* > 0.05; after: *X*^2^(1, *N* = 4,654) = *X*, *P* < 0.05].

At both intersections, during both precipitation and non-precipitation periods (718 and 8,474 images in the residential sample, 592 and 9,881 images in the commercial sample, respectively), there was no relationship between the number of pedestrians and bicyclists and crosswalk enhancement.

## Discussion

The results of this study indicate that two webcams in Washington, DC, USA were able to capture pedestrian and bicyclist activity before and after the enhancement of two crosswalks, and across a range of temperatures. Pedestrian and bicyclist annotation was not significantly different before and after the crosswalk improvement at either location.

An improved crosswalk may signal to drivers that there are non-drivers present, including walkers and cyclists. Therefore, it is unclear why pedestrian and bicyclist annotation did not increase after crosswalk improvement. Potential explanations include increased vehicular traffic due to other improvements, or unsafe crosswalks along the way to the improved crosswalks (29). Furthermore, it is possible that a change in pedestrians and bicyclists would have been detectable if the study had included a different time frame; 5 months elapsed between the crosswalk improvement and post-data. This may have missed an early, novelty effect, or may be too short of a time period for behavior change. Future research could include a more broad analysis of a network of crosswalks as well as a more broad study time frame. Such research may help explain variations in pedestrian presence in crosswalks after improvements, as well as establish which types of improvements are associated with the greatest increase in pedestrian activity over time.

Webcam images reflected weather-related differences in pedestrian’s activity. Fewer pedestrians were annotated in images captured when temperatures were cold or hot, or when precipitation was detected. At both intersections after the enhancement, more images contained pedestrians captured during non-normal temperatures compared to the year prior. At the commercial intersection, the relationship between pedestrian presence and non-ideal temperatures prior to the crosswalk enhancement was significant; following the improvement of the crosswalk, the relationship was not significant. At the residential intersection, the relationship between bicyclist presence and precipitation prior to crosswalk enhancement was significant; following the improvement, the relationship was not significant. These suggest that the crosswalk may have played a larger influence on pedestrian and bicyclist presence than ambient temperature or precipitation. Or stated another way, the addition of the crosswalk diminished the change in pedestrians between ideal temperatures and non-ideal temperatures, and the change in bicyclists between precipitation and non-precipitation. This may be due to an increased sense of pedestrian or bicyclist safety or speed in crossing when temperatures were less than ideal.

In this study, sufficient hourly precipitation data were not accessible. Researchers attempted to identify precipitation visually using MTurk workers. The collection of reliable precipitation annotation by MTurk workers in images was a challenge. Trained research assistants were capable of identifying precipitation, but a considerable amount of resources are required to sustain research assistants. Therefore, while the results indicate that reliable precipitation data may be collected from publicly available webcam images, researchers should continue to develop and validate weather-related image questions for crowdsourcing tasks, or incorporate crowdsource worker training. Other options include linking temperature, precipitation, and other weather-related data to webcam databases such as AMOS, making that information instantly accessible to relevant stakeholders. The presence of pedestrian and bicyclist activity during inclement weather are of interest to community stakeholders invested in safety and transportation (12).

Proximity to a built environment intervention, such as a crosswalk addition, does not necessarily indicate an impact will be made on amount of active transportation (7, 13). Webcams could be used to examine the influence of built environment changes on specific population groups such as adolescents or older adults. These populations generally have different motivating factors for participation in active transportation and may receive more benefits from tailored built environment features than the general population (1, 3, 5, 6, 8, 17, 18, 20, 30).

Future webcam research should include the simultaneous analysis of multiple (e.g., greater than two) webcam locations in order to establish the external validity of the method. Future webcam research should also include long-term follow-up of built environment changes. Studies should not be restricted to crosswalk enhancement or bike lane addition (14), but could include speed bump additions, median enhancements, or other environmental improvements relevant to specific communities. There is also the opportunity to use this as part of a mixed-methods approach, or in conjunction with civic and community partners in identifying non-built pedestrian safety improvement efforts, such as neighborhood watch groups and speed limit reductions.

### Limitations

Limitations of the present analyses include the use of only two intersections. The images used for analyses only provide information on behaviors at two specific locations, restricting the external validity of the findings. This study was unable to determine whether or not pedestrians were changing their routes or to capture a sense of safety prior to and following the enhancement of crosswalks. There are also limitations to using webcams for research. These limitations have been documented elsewhere (22), and include occlusion of pedestrians and bicyclists due to physical obstructions, weather-related obstructions, and camera-related issues (e.g., missing and unstable images). A final limitation could include the categorization of temperatures into ideal and non-ideal. Categorization was based on the average temperature recorded during image capture. Therefore, more nuanced differences which could have resulted from more in-depth temperature categorization may not have been discovered.

## Conclusion

Despite these limitations, the ubiquity and unobtrusive nature of webcams presents an opportunity to understand the effects of a variety of built environment improvements, across time and environments, in a cost-effective manner. While the applications of this method are still being fully developed, there is great promise in its potential. Possible applications include understanding which populations are benefiting from built environment enhancements, as well as broader studies examining the synergistic effects of multiple built environment changes.

The use of webcams and crowdsourcing is a promising technique for evaluating the effects of built environment interventions and environmental factors, such as temperature, on active transportation. As the method continues to develop, it is crucial that researchers and practitioners across community health and planning fields collaborate to explore various environments, interventions, and healthy behaviors.

## Author Contributions

JH contributed to the conceptualization of the study and manuscript revision; AM analyzed data and drafted the manuscript; AB assisted with data analysis and manuscript drafting; AS assisted with creation of the crowdsourcing task, data collection, and manuscript revision; and RP contributed to study design and manuscript revision.

## Conflict of Interest Statement

The authors declare that the research was conducted in the absence of any commercial or financial relationships that could be construed as a potential conflict of interest.

## Footnotes

^1^http://amos.cse.wustl.edu/

^2^https://www.mturk.com

^3^http://amos.cse.wustl.edu/submit_camera

^4^http://amos.cse.wustl.edu/camera?id=919

^5^http://amos.cse.wustl.edu/camera?id=942

^6^http://www.ncep.noaa.gov/
